# Next generation sequencing-aided screening, isolation, molecular identification, and antimicrobial potential for bacterial endophytes from the medicinal plant, *Elephantorrhiza elephantina*

**DOI:** 10.3389/fmicb.2024.1383854

**Published:** 2024-05-22

**Authors:** Matsobane Tlou, Benedict Ndou, Nokufa Mabona, Adivhaho Khwathisi, Collins Ateba, Ntakadzeni Madala, Mahloro Hope Serepa-Dlamini

**Affiliations:** ^1^Department of Biochemistry, School of Physical and Chemical Sciences, North-West University, Mmabatho, South Africa; ^2^Department of Biochemistry and Microbiology, University of Venda, Thohoyandou, South Africa; ^3^Department of Microbiology, Faculty of Natural and Agricultural Sciences, School of Biological Sciences, North-West University, Mmabatho, South Africa; ^4^Department of Biotechnology and Food Technology, University of Johannesburg, Doornfontein Campus, Johannesburg, South Africa

**Keywords:** *Elephantorrhiza elephantina*, next-generation sequencing, taxonomy, bacterial endophytes, phylogeny, secondary metabolites, antimicrobial activity

## Abstract

*Elephantorrhiza elephantina*, a wild plant in southern Africa, is utilized in traditional medicine for various ailments, leading to its endangerment and listing on the Red List of South African Plants. To date, there have been no reports on bacterial endophytes from this plant, their classes of secondary metabolites, and potential medicinal properties. This study presents (i) taxonomic characterization of bacterial endophytes in leaf and root tissues using 16S rRNA, (ii) bacterial isolation, morphological, and phylogenetic characterization, (iii) bacterial growth, metabolite extraction, and LC–MS-based metabolite fingerprinting, and (iv) antimicrobial testing of bacterial crude extracts. Next-generation sequencing yielded 693 and 2,459 DNA read counts for the rhizomes and leaves, respectively, detecting phyla including Proteobacteria, Bacteroidota, Gemmatimonadota, Actinobacteriota, Verrucomicrobiota, Dependentiae, Firmicutes, and Armatimonodata. At the genus level, *Novosphingobium*, *Mesorhizobium*, *Methylobacterium*, and Ralstonia were the most dominant in both leaves and rhizomes. From root tissues, four bacterial isolates were selected, and 16S rRNA-based phylogenetic characterization identified two closely related *Pseudomonas* sp. (strain BNWU4 and 5), *Microbacterium oxydans* BNWU2, and *Stenotrophomonas maltophilia* BNWU1. The ethyl acetate:chloroform (1:1 v/v) organic extract from each isolate exhibited antimicrobial activity against all selected bacterial pathogens. Strain BNWU5 displayed the highest activity, with minimum inhibitory concentrations ranging from 62.5 μg/mL to 250 μg/mL against diarrhoeagenic *Escherichia coli*, *Escherichia coli O157:H7*, *Salmonella enterica*, antibiotic-resistant *Vibrio cholerae*, *Staphylococcus aureus*, *Bacillus cereus*, and *Enterococcus durans*. LC–MS analysis of the crude extract revealed common antimicrobial metabolites produced by all isolates, including Phenoxomethylpenicilloyl (penicilloyl V), cis-11-Eicosenamide, 3-Hydroxy-3-phenacyloxindole, and 9-Octadecenamide.

## Introduction

The plant microbiome comprises a group of commensal microbes that are classified into epiphytic, phyllospheric, rhizospheric, and endophytic based on their association with the plant host. The microbiome is influenced by complex interactions between the hosts and environmental factors such as salinity, soil-type, −structure, −moisture, −organic matter and root exudates (Fierer, 2017). The endophytes are communities of microorganisms, including bacteria, fungus, or actinomycetes that spend all or part of their life cycles inside the tissues of a plant while often presenting no disease symptoms (Arora and Ramawat, 2017). They are ubiquitous in nature and exhibit complex interactions with their hosts, which may involve mutualism, antagonism and rarely parasitism (Nair and Padmavathy, 2014; Gouda et al., 2016).

Endophytic microbes exhibit two distinct categories: obligate, characterized by their uncultivability and reliance solely on the host for survival and growth; and facultative survivors, which are culturable and can operate independently from the host. Despite the extensive history on microbial culture and enumeration, the uncultivability of obligate endophytes presents a bottleneck in the identification and measurement of endophyte diversity and community structure in plants (Gaiero et al., 2013). The paradigm that only 1% of microbes for a given environment is culturable has had a profound impact on our understanding of microbial ecology and is still a major motivation for using molecular tools for studying microbial communities (Martiny, 2019). However, there has been substantial progress in cultivation techniques which suggest that the paradigm may have shifted (Martiny, 2019).

Medicinal plants are wild plant species that have been used for centuries in traditional or herbal medicine for the treatment of various diseases. According to the International Union for Conservation of Nature and the World Wildlife Fund, over 15,000 flowering medicinal plants face the threat of extinction, primarily because of overharvesting and habitat destruction driven by the growing global population and increased demand for plant-based resources (Chen et al., 2016). *Elephantorrhiza elephantina* is a member of a small and purely African genus represented by nine plant species on the continent (Glen, 2003). It is an important plant resource in southern Africa, where the tuberous rhizome it is used as food and medicine by the Indigenous people (Coates and Coates, 2002). The underground rhizomes are one of the primary herbal medicines in southern Africa and consequently sold in various traditional medicine markets (Williams et al., 2001; Mander et al., 2006). The ethnomedicinal uses for this plant include the treatment of ailments such as diarrhea, diabetes, chest complaints, heart conditions, hypertension, syphilis, infertility in women, bladder problems, skin problems, waist pain in infants, fever, and hemorrhoids (Semenya and Potgieter, 2013; Lall and Kishore, 2014; Maroyi, 2017). Due to the unsustainable use of *E. elephantina* rhizomes driven by the demand, the plant is considered endangered and has been included on the Red List of South African Plants by the South African National Biodiversity Institute (Williams, 2008).

Some of the ethnomedical properties associated with *E. elephantina* have been corroborated by the pharmacological activities reported for this plant, i.e., antifungal (Mukanganyama et al., 2011), antibacterial (Mpofu S. et al., 2014), anthelmintic (Maphosa et al., 2010), antiplasmodial (Clarkson et al., 2004), anti-rickettsial (Naidoo et al., 2005), antioxidant, (Mpofu S. J. et al., 2014) and anti-inflammatory activities (Maphosa et al., 2009). Furthermore, *E. elephantina* is known to produce a wide array of secondary metabolites/bioactive metabolites including anthocyanidins, anthraquinones, esters, phenolic compounds, flavonoids, glycosides, phytosterols, saponins, tannins, and triterpenoids (Msimanga et al., 2013; Maroyi, 2017), which can be linked to the reported pharmacological activities. However, in 2015, Ludwig-Müller highlighted the need to re-evaluate the well-known plant production systems based on the findings that interesting metabolites could be produced by endophytes instead of the host. The anticancer drug, taxol is a prominent example of plant-derived metabolite synthesized by fungal endophyte, *Taxomyces andreanae* which was isolated from the plant, *Taxus brevifolia* (Strobel et al., 1993). The search for taxane producing endophytes was prompted by the high demand for the drug and consequently, the search for alternative sources to the plant material (Ludwig-Müller, 2015). The production of plant-derived metabolites has also been reported for endophytic bacterial isolates (Trapp et al., 2015; Photolo et al., 2020) and this, further underscores a report by Chatterjee et al. (2019), which highlighted that the biologically active compounds are frequently not the plant’s own metabolic products but are instead produced by the endophytes.

To date, there have been no reports on the plant microbiome of *E. elephantina*. Consequently, our understanding of the nature of endophytes that inhabit these plants and the interactions between the host plant and various classes of microbes remains limited. Furthermore, it is deducible from literature that, the high demand for the rhizome implies that the plant is often uprooted and destroyed during rhizome harvesting, hence the endangerment of the plant species. This study hypothesized that, akin to all plants, the internal tissues of *E. elephantina* harbor various forms of endophytes, including bacteria. The secondary metabolites produced by the endophytic bacteria are also likely to influence the diverse pharmacological activities associated with the plant and therefore, the bacteria can be explored as possible alternative sources for the desired biological activity. Moreover, the characteristic rapid growth on relatively inexpensive culture media coupled to the ease of genetic manipulation *in vitro* indicates that bacterial endophytes can be useful as easily accessible and sustainable producers of natural products (Gouda et al., 2016). Therefore, in this study we report on 16S rRNA-based screening of the bacterial metagenome of the leaf and rhizome tissue using next-generation sequencing and the relative taxonomic classification of bacterial populations from both tissues. The study also reports on the culture-based isolation of bacterial endophytes from the rhizome, phylogenetic classification, the production of bioactive metabolites and antimicrobial activity assays.

## Materials and methods

### Plant sample collection and surface sterilization

Healthy disease-free plant samples (roots and leaves) were collected (Date: 22/03/2022) from *E. elephantina*, in Mahikeng (25°57′ 27,5”S 25°26′30,6″E), North West province, South Africa. The identification of the plant and sample collection was performed under the guidance of Dr. Madeleen Struwig (S.D. Phalatse Herbarium, North-West University, Mafikeng Campus). The plant samples were thoroughly washed with tap water to remove soil, cut into small segments, treated with Tween 80 for 10 min (min) with vigorous shaking and then rinsed with distilled water. The plant samples were further immersed in 70% ethanol for 1 min and then treated with 1% NaOCl for 10 min. The samples were then rinsed five times with sterile distilled water and the final wash was plated on Luria Bertani (LB) agar plates (g/L; peptone 10, yeast extract 5, sodium chloride 10, and agar 15, pH 7.0) as a control.

### Metagenomics analysis

The surface sterilized leaf and rhizome samples were homogenized in Phosphate-Buffered Saline (PBS) and 10 mL of each sample sent to Inqaba Biotechnical Industries (Pretoria, South Africa) a commercial service provider, for bacterial metagenome analysis. Briefly, bacterial genomic DNA was extracted from the plant samples using the Quick-DNA™ Fungal/Bacterial Miniprep Kit. The extracted DNA samples were used as a template in a PCR using a universal primer pair 27F (5′-AGAGTTTGATCTGGCTCAG-3′) and 1492R (5′-AAGGAGGTGWTCCARCC-3′) targeting the V1 -V9 region of the bacterial 16S rRNA gene (Huse et al., 2008). The following conditions were used for polymerase chain reaction (PCR): 1X initiation cycle at 92°C for 2 min, 30X denaturation cycles at 92°C for 30 s, 30X primer annealing cycles at 52°C for 30 s, 30X extension cycles at 72°C for 2 min, and a 1X elongation cycle at 72°C for 2 min. The resulting amplicons were barcoded with Pacbio M13 barcodes for multiplexing through limited cycle PCR.^1^ The resulting barcoded amplicons were quantified and pooled with equimolar and then AMPure PB bead-based purification step was performed. The PacBio SMRTbell library was prepared from the pooled amplicons following the manufacture protocol. Sequencing primer annealing and polymerase binding were done following SMRTlink Link software protocol to prepare the library for sequencing on PacBio Sequel IIe system. Raw subreads were processed through the SMRTlink (v10.2) Circular Consensus Sequences (CCS) algorithm to produce highly accurate reads (>QV40). These highly accurate reads were processed through DADA2^2^ and qiime2^3^ for quality control assessment and taxonomic classification, respectively.

### Isolation of bacterial endophytes and gram staining

The bacterial endophytes were isolated from the plant samples using a method described by Jasim et al. (2014). Briefly, the outer surface of the plant material was trimmed off and the sample homogenized in PBS (g/L; sodium chloride 8, potassium chloride 0.2, disodium hydrogen phosphate 1.44 and potassium dihydrogen phosphate 0.24, pH 7.4). The samples were serially diluted up to 10^−3^ and 0.1 mL of the dilution plated on LB agar (g/L; peptone 10, yeast extract 5, sodium chloride 10, and agar 15, pH 7.0) and incubated at 30°C for a few days. The plates were observed daily for bacterial colony growth followed by isolation and sub-culturing in LB agar until pure colonies were obtained. Glycerol stocks (50%, glycerol diluted in sterile LB broth) of each bacterial isolate were prepared and stored at −80°C for future use. Afterwards, the pure colonies were subjected to Gram staining as described by Collins et al. (2004) to establish morphological characteristics such as shape and Gram stain reaction. Gram stain slides were observed using an OLYMPUS CH20BIMF200 compound bright-field microscope (Lasec® Group, Cape town, South Africa) with 100x magnification.

### Genomic DNA extraction, 16S rRNA PCR and DNA sequencing

Genomic DNA was extracted from an overnight starter culture using Quick-DNA™ Fungal/Bacterial Miniprep Kit following the manufacturer’s protocol. The extracted genomic DNA was then quantified using a NanoDrop™ ND-2000 UV–vis spectrophotometer (Thermo Fisher Scientific Inc., Waltham, USA). The 16S rRNA gene for each isolate was amplified according to a method described by Hongoh et al. (2003). The PCR was performed using the universal primers 27F (5′-AGAGTTTGATCTGGCTCAG-3′) and 1492R (5′-AAGGAGGTGWTCCARCC-3′) which were purchased from Inqaba Biotechnological Industries (Pretoria, South Africa). PCR was in 25 *μ*L total volumes using the following conditions: 1X initiation cycle at 92°C for 2 min, 30X denaturation cycles at 92°C for 30 s, 30X primer annealing cycles at 52°C for 30 s, 30X extension cycles at 72°C for 2 min, and a 1X elongation cycle at 72°C for 2 min followed by termination at 4°C. The PCR products were analyzed on a 1% agarose gel by electrophoresis at a constant 100 V and 200 mA. The positive products were then excised from the gel and purified u + sing the GeneJet gel extraction kit. The purified PCR products were sequenced at Inqaba Biotechnical Industries (Pretoria, South Africa).

### Phylogenetic analysis

The CLC Bio Main Workbench was used to assemble the forward and reverse sequencing reads to form a consensus sequence for each isolate. This was followed by a Basic Local Alignment Search Tool (BLAST) search on the National Center for Biotechnology Information (NCBI) GenBank nucleotide sequence database^4^ to determine if a sequence in the database matches the query sequence (Altschul et al., 1997). Sequences of species closely matching the BLAST query to each sample along with their closely related taxa were obtained for the phylogenetic analysis. The sequences were aligned using the multiple sequences alignment tool, Clustal X 2.1 version (Thompson et al., 1994). The phylogenetic and molecular evolutionary analysis was conducted with MEGA X using maximum likelihood method with 1,000 bootstrap replicates (Kumar et al., 2018).

### Bacterial growth analysis and extraction of secondary metabolites

A starter culture was prepared by inoculating a single colony of the bacterial sample into 5 mL of nutrient broth (NB) and incubating it overnight at 37°C with continuous agitation at 120 rpm. Subsequently, 1 mL of the starter culture was transferred into 50 mL of LB broth (g/L; tryptone 10, yeast extract 5, and sodium chloride 10, pH 7.0) and cultivated at 37°C with continuous agitation at 120 rpm. Optical density readings were recorded in triplicate every 2 h (h) over a 48 h period using the ONDA (UV/VIS) spectrophotometer (Lasec® Group, Cape town, South Africa) at a wavelength of 600 nm.

The extraction of endophytic bacterial secondary metabolites was carried out using the method previously described in Balachandran et al. (2012) with minor modifications. Briefly, LB broth (1 L) was prepared in 2 L Erlenmeyer flasks and autoclaved at 121°C for 20 min. Each of the 2 L flasks was inoculated with the isolates and incubated at 37°C for 7 days agitating at 120 rpm. The cells were harvested, centrifuged at 10000 rpm for 15 min for biomass removal and equal volumes of ethyl acetate and chloroform (1,1 v/v) were added to the supernatant followed by vigorous agitation. The organic solvent layer was collected in a boiling flask, and it was further concentrated using a vacuum rotary evaporator (Lasec® Group, Cape town, South Africa) at 40°C. The crude extracts were transferred to 5 mL sterile vials and left to dry at room temperature ± 25°C.

### Antimicrobial assay

Minimum inhibitory concentration (MIC) studies of the crude extracts from the isolates were conducted following the method described by Andrews (Photolo et al., 2020) with minor modifications. Briefly, stock solutions were prepared by dissolving 1 mg of the extract in 1 mL of dimethyl sulfoxide (DMSO) to a final concentration of 1 mg/mL. These stock solutions were then serially diluted to concentrations of 500, 250, 125, 62.5, 31.25, 15.625, 7.813, 3.906, and 1.953 μg/mL using tryptic soy broth (TSB) (g/L; casein peptone (pancreatic) 17, dipotassium hydrogen phosphate 2.5, glucose 2.5, sodium chloride 5, and soya peptone (papain digest) 3). The pathogenic test strains used included Gram-negative bacteria diarrhoeagenic *Escherichia coli* DEC-NWU (GenBank Ascension Number: CP121294), an environmental *Escherichia coli* O157:H7 strain (GenBank Ascension Number: JAVLRS000000000), *S. enterica* DSS_NWU (GenBank Ascension Number: CP123007), and antibiotic resistant *Vibrio cholerae* (GenBank Ascension Number: CP122254) and Gram-positive bacteria *Staphylococcus aureus* (ATCC 26923), *Bacillus cereus.* (ATCC 10876), and *Enterococcus durans* NWUTAL1 (GenBank Ascension Number: VMRQ00000000) (Foka et al., 2020). Using McFarland 0.5 standard, 50 *μ*L of each pathogen was inoculated in 15 mL of TSB and incubated at 37°C for 24 h. In a 96 well microtiter plate, 100 *μ*L of the pathogenic test strains were added horizontally and 100 *μ*L of the different dilutions of the crude extracts were added vertically starting from the highest to the lowest concentrations. This was done following aseptic techniques. Similarly, this was done with the positive controls of ampicillin and streptomycin and for the negative control, DMSO was used. The microtiter plate was sealed with parafilm and incubated at 37°C for 16–20 h. After incubation, 10 *μ*L of 4 mg/mL iodonitrotetrazolium chloride was added to each well. The MIC was recorded as the lowest concentration with clear wells, which indicated the absence of microbial growth. The antimicrobial experiments were performed in triplicates (*n* = 3).

### Metabolite identification using liquid chromatography mass spectrometry

The crude extracts were analyzed on a liquid chromatography–quadrupole time-of-flight tandem MS instrument (LCMS-9030 qTOF) (Shimadzu Corporation, Kyoto, Japan) using the method previously described in Ramabulana et al. (2021) with minor modifications. The chromatographic separation was performed on a Shim-pack Velox C18 column (100 mm × 2.1 mm with particle size of 2.7 μm) (Shimadzu Corporation, Kyoto, Japan) at 55°C. For all samples, an injection volume of 3 μL was used and run using a binary mobile phase gradient which consisted of solvent A: 0.1% formic acid in Milli-Q water and solvent B: Methanol with 0.1% formic acid. The flow rate was set to 0.3 mL/min throughout the set 20 min gradient with the following separation conditions: 5% B maintained for 3 min, 5–40% B over 3–5 min, 40–95% B over 5–12 min, from 12–16 min the conditions were maintained at 95% B, then the gradient was changed to 5% B between 16–18 min and maintained at 5% B for 2 min. The gradient was returned to initial conditions between 18–20 min which was followed by a 3-min column re-equilibration time. The chromatographic effluents were further analysed utilizing the qTOF high-definition mass spectrometer set to acquire negative electrospray ionization data. The subsequent parameters were set as: interface voltage of 4.0 kV, interface temperature of 300°C, nebulization, and dry gas flow 3 L/min, heat block temperature of 400°C, DL temperature of 280°C, detector voltage of 1.8 kV and the flight tube temperature at 42°C. Sodium iodide (NaI) was used as a calibration solution to monitor high mass accuracy. MS^1^ and MS^2^ (through data dependent acquisition) were generated simultaneously for all ions with an m/z range between 100–1,000 surpassing an intensity threshold of 5,000. Fragmentation experiments were performed using argon as a collision gas at a collision energy of 30 eV with a spread of 5 eV. Quality control (QC), pooled samples were used to condition the LC–MS system and for non-linear signal correction. The QC samples were injected at the beginning and end of the batch to ensure system equilibration. Furthermore, sample acquisition was randomized, and the QC sample analyzed every 10 injections to monitor and correct changes in the instrument response.

The SIRIUS software was used for the identification of metabolites (Dührkop et al., 2015). The SIRIUS software (version 4.9.12), was downloaded from Lehrstuhl Bioinformatik Jena website.^5^ Raw data obtained from the Shimadzu LCMS-9030 qTOF was converted to an open-source format (.mzML) prior to being uploaded to the SIRIUS software. To compute the molecular formulas, instrument type was set as qTOF, mass accuracy was set as 10 ppm and possible ionization was selected as [M–H]^+^. C, H, N, and O were selected for element searches, and the number of candidates was set to 10. Structure elucidation by CSI: FingerID was set to search in all databases and [M–H]^+^ as the only adduct (Dührkop et al., 2015). Canopus Class Prediction was also abled (Hoffmann et al., 2021).

### Statistical analysis

The data was analyzed using analysis of variance (ANOVA). The antimicrobial assay data were described as mean ± standard deviations (SD). This analysis was done using GraphPad prism 9 (Belayneh et al., 2022) and *p* values less than 0.05 were statistically different.

## Results

### Metagenome analysis, phylum and genus-level distribution of leaf, and rhizome bacterial communities

The NGS data analysis of the bacterial metagenome in *E. elephantina* yielded 693.0 read counts for the rhizome, revealing a diverse composition with 7 phyla, 11 classes, 18 orders, 21 families, and 23 genera (data not shown). Similarly, the leaves exhibited a total of 2,459 read counts, reflecting a rich biodiversity comprising 8 phyla, 8 classes, 12 orders, 17 families, and 19 genera (data not shown). The NGS data obtained for leaves and rhizome were deposited to the NCBI BioSample database and, respectively, assigned BioSample accessions SAMN40452947 and SAMN40452948.

**Figure 1:**
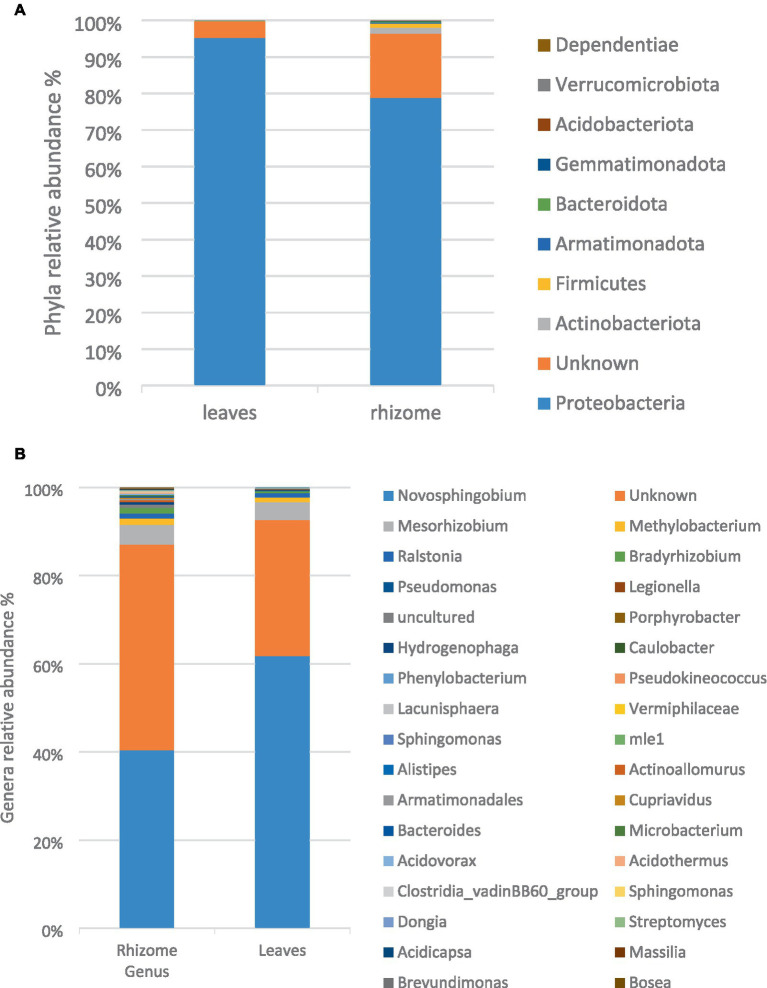
The relative taxonomic abundance of bacterial phyla **(A)** and genus **(B)** in leaves and rhizome samples based on analysis of full-length 16S gene amplicons.

At the phylum level, the bacterial composition in both leaves and rhizome of *E. elephantina* encompassed Proteobacteria, Bacteroidota, Gemmatimonadota, Actinobacteriota, Verrucomicrobiota, Dependentiae, Firmicutes, and Armatimonodata, with Proteobacteria predominating in both plant parts at 90 and 78% in the rhizome and leaves, respectively. Additionally, 17.6 and 4.51% of unidentified bacteria were noted in the rhizome and leaves, respectively. Notably, Verrucomicrobiota and Dependentiae phyla were exclusive to the leaves, while Armatimonadota phylum was solely detected in the rhizome (Figure 1A). Among the genera, Novosphingobium, Mesorhizobium, Methylobacterium, and Ralstonia, all belonging to the Proteobacteria phylum, dominated the microbiota in both leaves and rhizome. At the genus level, the rhizome exhibited greater bacterial diversity compared to the leaves (Figure 1B).

### Isolation of bacterial endophytes, morphological, and phylogenetic identification

The bacterial isolation experiment yielded 10 pure colonies designated as EER-1 to EER-10. To molecularly identify these bacterial isolates, the 16S rRNA gene was sequenced using universal primers 27F and 1492R and sequencing data revealed that EER-1, EER6, EER-9 and 10 exhibited a maximum identity of 99% or greater to specific reference strains, namely *Stenotrophomonas maltophilia*, *Microbacterium oxydans*, *Pseudomonas chengduensis*, and *Pseudomonas alcaligenes*, respectively (Table 1). Notably, EER-2 to EER-5 and EER-7 and 8 were either identified as mixed cultures or re-isolates and, consequently, were excluded from further consideration. The obtained 16S rRNA sequences were submitted to the National Center for Biotechnology Information (NCBI) and were assigned unique accession numbers, as detailed in Table 1.

**Table 1:** NCBI BLAST 16S rDNA gene sequences of bacterial endophytes isolated from *E. elephantina*.

Isolates	Assigned bacterial name	Gen bank accession number	NCBI results			
			Closest NBCI related bacterial species with accession number	Query coverage %	*E-*value	Identity similarity %
EE-R1	*Stenotrophomonas* sp. BNWU1	OQ859587	*Stenotrophomonas maltophilia* (OQ588742.1)	100%	0.0	99.57%
EE-R6	*Microbacterium* sp.BNWU2	OQ859588	*Microbacterium oxydans* (MT533951.1)	100%	0.0	99.76%
EE-R9	*Pseudomonas* sp. BNWU4	OQ859589	*Pseudomonas chengduensis* (MN099372.1)	100%	0.0	99.88%
EE-R10	*Pseudomonas* sp. BNWU5	OQ859590	*Pseudomonas alcaligenes* (MT323223.1)	99%	0.0	99.88%

**Figure 2:**
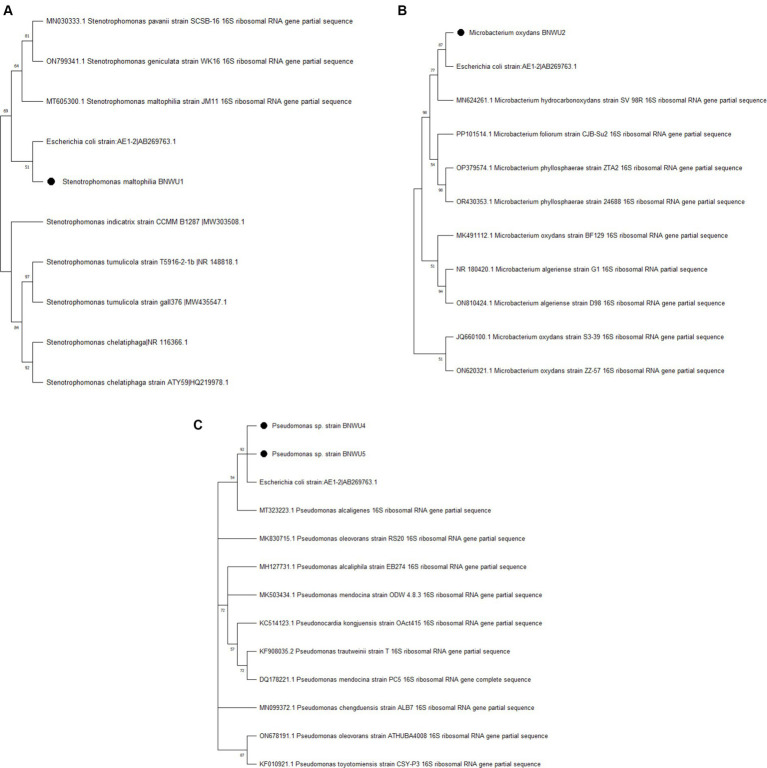
Maximum likelihood phylogenetic tree based on 16S rRNA gene sequence for BNWU1 **(A)**, BNWU2 **(B)**, and BNWU 4 and 5 **(C)** versus selected bacterial species from GenBank. Numbers above or below the nodes indicate bootstrap values generated after 1,000 replications.

The phylogenetic analysis of the 16S rRNA sequence of the isolates along with the sequences retrieved from the NCBI was carried out with MEGA X using the maximum likelihood method with 1,000 bootstrap replicates. The results showed that all the endophytic bacterial isolates grouped with various closely related bacterial species. Each genus was analyzed in separate phylogenetic trees (Figure 2) and it was observed that, *Stenotrophomonas* sp. BNWU1 (Figure 2A) has a monophyletic relationship with *Stenotrophomonas maltophilia* strain JM11, *S. pavanii* strain SCSB and *S. geniculate* strain WK16 supported by 48% bootstrap value. It also formed a separate clade indicating it could be different from the other 3 *Stenotrophomonas* species. *Microbacterium* sp. BNWU2 (Figure 2B) had a paraphyletic relationship with other *Microbacterium* species and formed a separate clade. On the other hand, *Pseudomonas* sp. BNWU4 and BNWU5 both formed separate clades, indicating they are different species, and both formed paraphyletic relationship with other *Pseudomonas* species (Figure 2C).

Gram staining was employed to assess the morphological characteristics and Gram stain reactions of the bacterial isolates. The results revealed that all isolates exhibited a rod-shaped or bacilli morphology and displayed a Gram-negative staining pattern, except for BNWU2, which exhibited a Gram-positive staining reaction (data not shown).

### Bacterial growth and metabolite extraction

The bacterial isolates were cultured in LB broth for a duration of 48 h to track the growth dynamics over time. Notably, uniform growth profiles were observed for the *Pseudomonas* isolates (BNWU4 and 5). Additionally, it was observed that the *Pseudomonas* isolates entered the stationary phase of growth within the initial 12 h post-inoculation, while BNWU1 and BNWU2 reached this phase after 24 h (data not shown). The stationary growth phase was selected for the extraction of secondary metabolites because various biological activities, are typically produced during this phase in a fermentation medium after the completion of microbial growth (Seyedsayamdost, 2019).

### Antimicrobial assay

The chloroform: ethyl acetate extracts from all isolates were tested for antibacterial activity against seven pathogenic strains: Gram-negative bacteria *Escherichia coli* (ATCC25922), *E. coli* 0.157:H7 (*Salmonella* sp., and *Vibrio cholerae*); Gram-positive bacteria *Staphylococcus aureus*, *Bacillus* sp., and *Enterococcus durans*. The MIC for the crude extracts for all isolates ranged from 62.5 μg/mL to 250 μg/mL. with BNWU5 recording the lowest MIC values/high antimicrobial activity when compared to other isolates (Table 2). When the endophytic bacteria’s crude extracts were tested against *Salmonella* sp., the endophytic bacterial crude extracts showed lower MIC values (250 μg/mL) than the positive controls (ampicillin) (Table 2). The lowest MIC value was 62.5 *μ*g/mL which was observed for *Pseudomonas* sp. Strain BNWU5 against pathogenic strain *Bacillus* sp. (Table 2).

**Table 2:** MIC values of antimicrobial activities for endophytic bacterial extracts.

Bacterial Strain	BNWU1 MIC (μg/mL)	BNWU2 MIC (μg/mL)	BNWU4 MIC (μg/mL)	BNWU5 MIC (μg/mL)	Ampicillin MIC (μg/mL)	Streptomycin MIC (μg/mL)
*Staphylococcus aureus* (ATCC26923)*	250	250	250	125	62.5	nt
*Escherichia coli* 0.157 (ON645905)*	250	250	125	125	15.625	nt
*E. coli* (ATCC25922)	250	250	250	125	15.625	nt
*Salmonella* sp.	250	250	250	250	nt	500
*Bacillus* sp.	250	125	125	62.5	125	nt
*Enterococcus durans*	250	125	250	125	15.625	nt
*Vibrio cholerae*	125	125	250	125	nt	62.5

^*^nt, Not Tested.

### Metabolite identification

To unravel the compounds contributing to the observed antimicrobial activity or antioxidant properties, LC-qTOFMS analysis was conducted. The identification of these compounds was based on their retention times (RT), parent masses, and molecular formulas, as outlined in Table 3 and Supplementary Figures S1A–D. The LC-qTOFMS analysis revealed a diverse array of bioactive compounds within the bacterial endophyte extracts. This included a range of fatty acids, alkaloids, flavonoids, peptides, and various phenolic compounds. The presence of these chemical classes is noteworthy, as they have established associations with significant antimicrobial and antioxidant properties, aligning with the observed effects of the bacterial endophyte extracts. The comprehensive identification of these compounds paves the way for a deeper understanding of the molecular basis of the observed bioactivity and opens avenues for further exploration of these endophytic bacterial extracts for potential therapeutic applications. Refer to Table 3 for detailed information on the identified compounds.

**Table 3:** Bioactive metabolites identified from the crude extracts for the bacterial isolates.

RT (min)	Parent mass	Molecular formula	Name of the compound	Biological activity	Bacterial endophyte	References
Alkaloids						
2.02	251.15	C12H18N4O2	Cyclo (histidyl-leucyl)	Antioxidant activity	EER1, EER10	Furukawa et al. (2012)
4.95	188.07	C_11_H_9_NO_2_	Indoleacrylic acid	Antioxidants, anti-inflammatory properties	All	Wlodarska et al. (2017)
5.57	217.10	C_12_H_12_N_2_O_2_	L-Abrine	Anticancer, antioxidant properties	EER9	Laskar et al. (2019)
12.51	250.09	C16H13NO3	3-Hydroxy-3-phenacyloxindole	Antimicrobial, anticancer, and anti-inflammatory properties.	All	Barupal and Fiehn (2019)
12.50	250.09	C_16_H_11_NO_2_	Cinchophen	Antimicrobial	EER1, EER6, EER9	Ezelarab et al. (2023)
7.63	190.09	C_11_H_11_NO_2_	methyl 2-(1H-indol-3-yl)acetate	antineoplastic agent	EER6, EER9	Zhang et al. (2006)
Flavonoids						
12.82	311.16	C_20_H_22_O_3_	Avobenzone	Antioxidants, Anti-aging, Anti-radiation activity	All	Gamit et al. (2023) and Gholap et al. (2023)
12.50	379.24	C_24_H_27_NO_2_	Octocrylene	Antioxidant activity, Anti-aging, Anti-radiation activity	All	Li et al. (2018) and Saboon et al. (2023)
11.55	609.16	C_31_H_28_O_13_	epicatechin-(4beta- > 8)-4’-O-methylgallocatechin	Wound healing activity, anticancer, antioxidant and anti-inflammatory	All	Schmidt et al. (2011) and Ramalingam et al. (2022)
Lipids						
14.15	473.40	C31H52O3	Alpha-Tocopherol Acetate (Vitamin E acetate)	Antioxidant, neuroprotective, antiviral, immunomodulatory, and anti-proliferative	EER6	Yang and McClements (2013)
13.02	563.56	C_18_H_35_NO2	9-Octadecenamide	Antimicrobial	All	Dos Reis et al. (2019)
13.59	310.31	C20H39NO	cis-11-Eicosenamide	Antimicrobial activity, Biocontrol	All	Qi et al. (2022)
12.96	881.76	C_55_H_102_O_6_	1-Stearoyl-2-oleoyl-3-palmitoleoyl-glycerol		All	
Peptides						
1.32	221.09	C_11_H_12_N_2_O_3_	5-hydroxy-D-tryptophan		All	
0.343	756.39	C_43_H_53_N_3_O_9_	Beauvericin G2	Anticancer, Anti-inflammatory, Antimicrobial activity	ERR10	Xu et al. (2007) and Wu et al. (2018)
4.73	279.13	C_14_H_18_N_2_O_4_	Tyrosylproline	Antibacterial	EER9 & EER10	Wattana-Amorn et al. (2016)
5.08	384.11	C14H17N5O8	Succinyladenosine		All	
2.57	369.11	C_16_H_20_N_2_O_6_S	Phenoxomethylpenicilloyl (penicilloyl V)	Antimicrobial activity	All	WHO et al. (2009)
Phenolic compounds						
11.24	279.16	C16H22O4	Dibutyl Phthalate	Antifungal, antibacterial, antiviral, and antioxidant activities	All	Khatiwora et al. (2012) and Shobi and Viswanathan (2018)
5.76	261.12	C_14_H_16_N_2_O_3_	Maculosin	Antioxidant, anti-cancer, and non-toxicity	All	Paudel et al. (2021)

## Discussion and conclusion

This study provides initial insights into the endophytic bacterial community residing in the leaves and roots of the medicinal plant *E. elephantina*. The examination of leaves and rhizome microbiome revealed a diverse composition of phyla, namely Proteobacteria, Bacteroidota, Gemmatimonadota, Actinobacteriota, Verrucomicrobiota, Dependentiae, Firmicutes, and Armatimonodata, with Proteobacteria being the predominant phylum in both tissues. The prevalence of this phylum in the plant samples is unsurprising and is aligned with observations in various environmental samples (Spain et al., 2009; Tkacz et al., 2018). Notably, some bacteria identified in the study were novel, showing no homologies to previously studied bacterial phyla in the databases. These findings lay the groundwork for future research focused on the isolation, characterization, and application of metabolite-producing endophytic bacteria from *E*. *elephantina*.

Given the notably higher bacterial diversity found in the rhizome compared to the leaves, the rhizome was selected for the isolation of bacteria. Subsequently, four isolates were selected and tentatively identified as, *Stenotrophomonas* sp. BNWU1, *Microbacterium* sp. BNWU2, and two *Pseudomonas* sp. BNWU4 and BNWU5 based on the 16S rRNA blast search results. The phylogenetic characterization revealed that both BNWU2, 4 and 5 are characterized by a paraphyletic relationship to closely related species while, BNWU1 shows a monophyletic relationship with other *Microbacterium* species. Therefore, in our study the ancestry for most of the isolates has not been unambiguously established and it represents a failure of resolution in the complete identification of the two isolates (Hoelzer and Meinick, 1994; Lewis et al., 2005). This could be due to thefact that the molecular marker, 16S rRNA is not infallible and there are reports to this effect (Boudewijns et al., 2006). Therefore, techniques such as the multilocus sequence analysis (MLSA) and whole genome sequencing can be very useful in the resolution of the identity of the isolates as demonstrated by Maela and Serepa-Dlamini (2023) and Diale et al. (2021).

All isolates can be categorized within the phylum Proteobacteria, except for *Microbacterium* BNWU2, which belongs to Actinobacteriota. Additionally, the NGS data accounted for genera Pseudomonas and Microbacterium indicating that the screening complemented the bacterial isolation method. However, Strenotrophomonas was not detected, and this may be due to the inherent limitation associated with using a single molecular marker in the resolution of closely related species (Poretsky et al., 2014) and the fact that samples were only collected once in this study. This is a first report of its kind albeit the lower diversity of bacterial isolates which may be due to the influence of culture conditions such as media and temperature (Ifeanyi et al., 2014). Moreover, it is widely acknowledged that metagenome analysis faces a limitation in discriminating between dead and viable cells, as highlighted by Tan et al. (2015). In the specific context of this study, it is crucial to recognize that the identification of bacteria through the screening technique does not always correlate with their cultivability. Nevertheless, employing various microbiological culture media, as suggested by Chauhan et al. (2020), holds the potential to enhance the diversity of bacterial isolates.

In this study, all the isolates were recorded to reach the stationary growth phase post incubation for 24-h. The decision to focus on this growth phase for secondary metabolite extraction was informed by the well-established tendency of bacteria during the stationary phase to produce secondary metabolites, including antibiotics and siderophores (Ruiz et al., 2010). Our analysis revealed a similar growth profile for the *Pseudomonas* species (BNWU4 and 5) when cultured in LB broth. This similarity suggests a genus-specific phenotype, reinforcing the notion of a close relationship between these isolates.

The antibacterial activity for the crude extracts obtained from all the bacterial isolates was determined against seven pathogenic strains: *Escherichia coli*, *E. coli* 0.157, *Salmonella* sp., *Vibrio cholera*, *Staphylococcus aureus* (ATCC26923), *Bacillus* sp., and *Enterococcus durans*. The MIC varied depending on the test strain used, the extracts showed interesting results as they were effective against all the test strains at MIC lower than 1 mg/mL. Crude extracts exhibiting a minimum inhibitory concentration of 1 mg/mL or lower are considered significantly active (Van Vuuren, 2008). This shows that endophytic bacteria from *E. elephantina* have great potential for the development of compounds containing bioactivities against human pathogenic microorganisms as shown in Table 3. The obtained results agree with other previous studies, that have proven endophytic bacteria are potential sources of novel bioactive compounds with antimicrobial activity (Makuwa and Serepa-Dlamini, 2021). Furthermore, the alkaloids, flavonoids, and phenolic compounds that were detected in this study have been previously reported to possess antimicrobial activities and antioxidant properties both *in vitro* and *in vivo* studies (Martins et al., 2016; Yin et al., 2016).

Bioactive compounds including cinchophen, 9-octadecenamide, tyrosylproline, beauvericin, and phenoxomethylpenicilloyl (penicilloyl V) (Table 3) which are known for antimicrobial activities were detected in this study. Beauvericin which was only detected in *Pseudomonas* sp. Strain BNWU5 and belongs to the enniatins antibiotic family, characterized by a cyclic hexadepsipeptide and the alternate presence of three d-hydroxy-isovaleryl and three N-methyl-phenylalanyl groups (Hamill et al., 1969). Caloni et al. (2020) reported that beauvericin has many biological activities such as antibacterial, antiviral, antifungal, antiparasitic, insecticidal and anticarcinogenic activities, and this may explain the high antibacterial activity of isolate BNWU5. Interestingly, the crude extracts from all the isolates tested positive for phenoxomethylpenicilloyl that is currently used as a prescription antibiotic for certain bacterial infections, such as streptococcal and pneumococcal upper respiratory tract infections, and the prevention of rheumatic fever and chorea (Rawson et al., 2021). Epicatechin-(4beta- > 8)-4’-O-methylgallocatechin a flavonoid that is known for wound healing (Schmidt et al., 2011; Ramalingam et al., 2022) activity was also identified in all the four isolates. Interestingly, similar/related compounds include epigallocatechin gallate, epicatechin gallate, and epicatechin have been reported from *E. elephantina* (Maroyi, 2017). Therefore, this supports the reports on endophytes that produce similar bioactive compounds as their host plant and therefore, can be further explored as alternative sources for the sought-after bioactivities (Deshmukh et al., 2020; Agrawal et al., 2022).

The robust antimicrobial properties observed in this study may also be attributed to the presence of various flavonoids, alkaloids, and phenolic compounds in the crude extracts, as revealed by our LC/MS findings (Table 3). Flavonoids and phenolic compounds are recognized for their ideal structural chemistry conducive to free radical scavenging activity (Kannan et al., 2016). Among the identified compounds, dibutyl phthalate, a phenolic compound, stood out. This compound has been reported to possess a spectrum of activities, including antifungal, antibacterial, antiviral, and antioxidant properties (Khatiwora et al., 2012; Shobi and Viswanathan, 2018). The antibacterial activity of dibutyl phthalate, as reported by Shobi and Viswanathan (2018), further supports its potential therapeutic applications. The previously mentioned studies on dibutyl phthalate focus on the identification and isolation of this metabolite from medicinal plant species. The recognition of this metabolite in the crude extract derived from the bacterial isolates in our study lends support to the idea that these compounds may originate from endophytic organisms rather than the metabolic processes of the host plant, as proposed by Ludwig-Müller (2015). Furthermore, numerous reports highlight instances where endophytes produce molecules initially attributed to plants. This underscores the growing understanding of endophytes as promising alternative sources for pharmaceutically active metabolites. Additionally, alkaloids and flavonoids, such as octocrylene, cyclo (histidyl-leucyl), avobenzone, indoleacrylic acid, and L-Abrine, were identified in our study. These compounds are known for their significant antimicrobial and antioxidant properties. Notably, avobenzone and octocrylene, recognized antioxidant agents, are commonly used in skin care products and cosmetics (Jesus et al., 2023). Indoleacrylic acid, identified in this study, emerges as a promising compound in drug discovery due to its multifaceted properties, including antibacterial, antioxidant, anti-inflammatory, and antidiabetic activities (Wlodarska et al., 2017; Liu et al., 2023). These findings underscore the diverse array of bioactive compounds present in the extracts from endophytic bacterial isolates, suggesting their potential as valuable sources for the development of antimicrobial drugs and antioxidant supplements.

Another exciting advancement in this study is the remarkable similarity observed in the LC–MS chromatograms (Supplementary Figures S1A–D) and chemical profiles of the secondary metabolites (Table 3) among the bacterial isolates. Several factors could account for this phenomenon: firstly, methodological nuances such as extraction and analytical techniques (Olivon et al., 2017). Secondly, the conservation of biosynthetic pathways, as demonstrated by Cimermancic et al. (2014). Thirdly, the possibility of horizontal gene transfer, wherein different bacterial strains acquire genetic material enabling the production of similar secondary metabolites, as discussed by Smillie et al. (2011). Lastly, the influence of common environmental factors cannot be overlooked. Bacteria residing in similar ecological niches or exposed to comparable environmental conditions may exhibit parallel adaptation strategies, leading to the production of comparable secondary metabolites, as suggested by Blin et al. (2013).

## Data availability statement

The datasets presented in this study can be found in online repositories. The names of the repository/repositories and accession number(s) can be found at: https://www.ncbi.nlm.nih.gov/genbank/, OQ859587, OQ859588, OQ859589, and OQ859590.

## Author contributions

MT: Writing – review & editing, Writing – original draft, Supervision, Resources, Project administration, Methodology, Funding acquisition, Formal analysis, Data curation, Conceptualization. BN: Writing – review & editing, Software, Methodology, Investigation, Data curation. NoM: Writing – review & editing, Software, Methodology, Investigation. AK: Writing – review & editing, Software, Methodology, Formal analysis. CA: Writing – review & editing, Resources, Methodology. NtM: Writing – review & editing, Software, Resources, Methodology, Investigation. MS-D: Writing – review & editing, Validation, Software, Methodology, Formal analysis.

## Footnotes

^1^ www.pacb.com

^2^ https://benjjneb.github.io/dada2/index.html

^3^ https://docs.qiime2.org/2021.11/

^4^ https://www.ncbi.nlm.nih.gov/genbank/

^5^ https://bio.informatik.uni-jena.de/software/sirius/
