# Supplementary material for: Next generation sequencing-aided screening, isolation, molecular identification, and antimicrobial potential for bacterial endophytes from the medicinal plant, Elephantorrhiza elephantina
Source: Front Microbiol. 2024 May 22;15:1383854. doi: 10.3389/fmicb.2024.1383854 (PMC11160484; doi:10.3389/fmicb.2024.1383854)
Supplement: Supplementary file 1 [file Presentation_1.pdf]

Supplementary Material

(A)

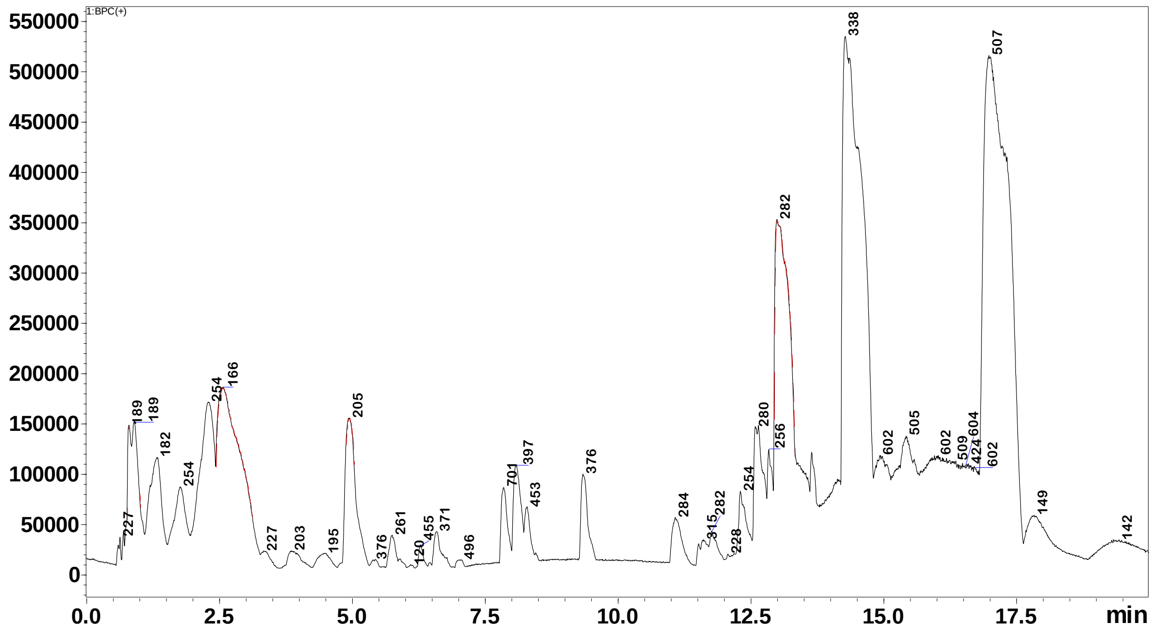

(B)

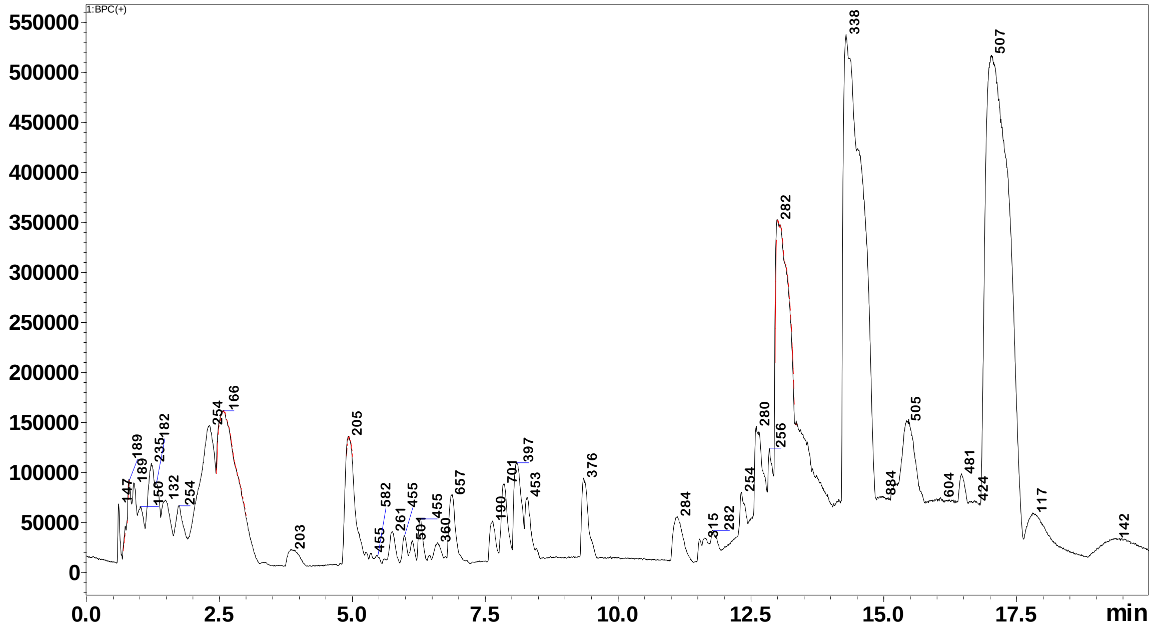

(C)

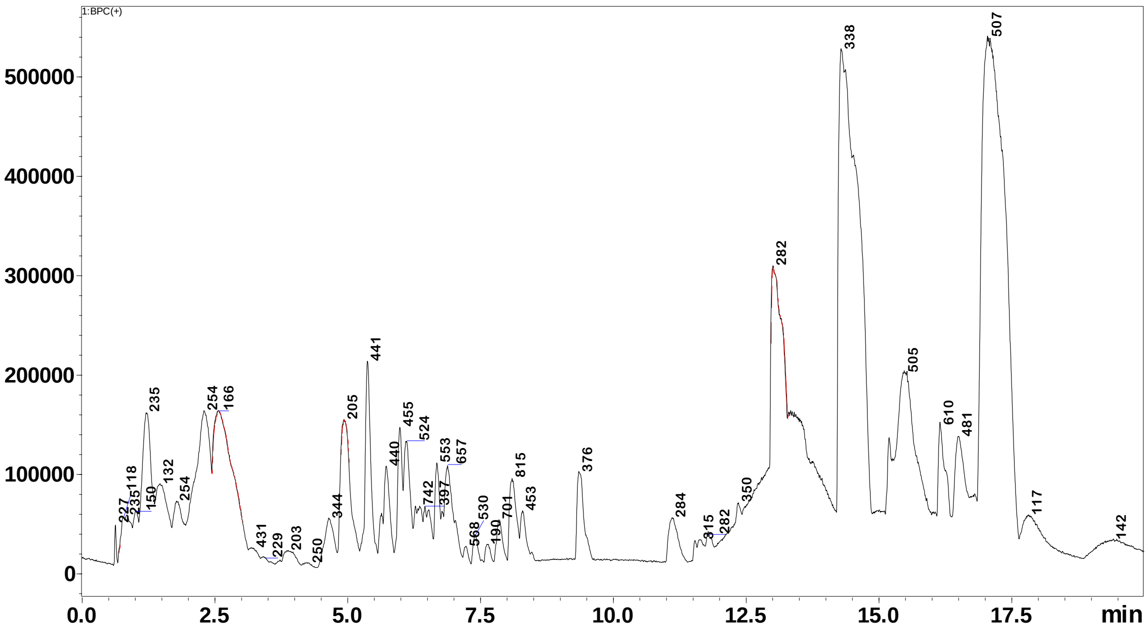

(D)

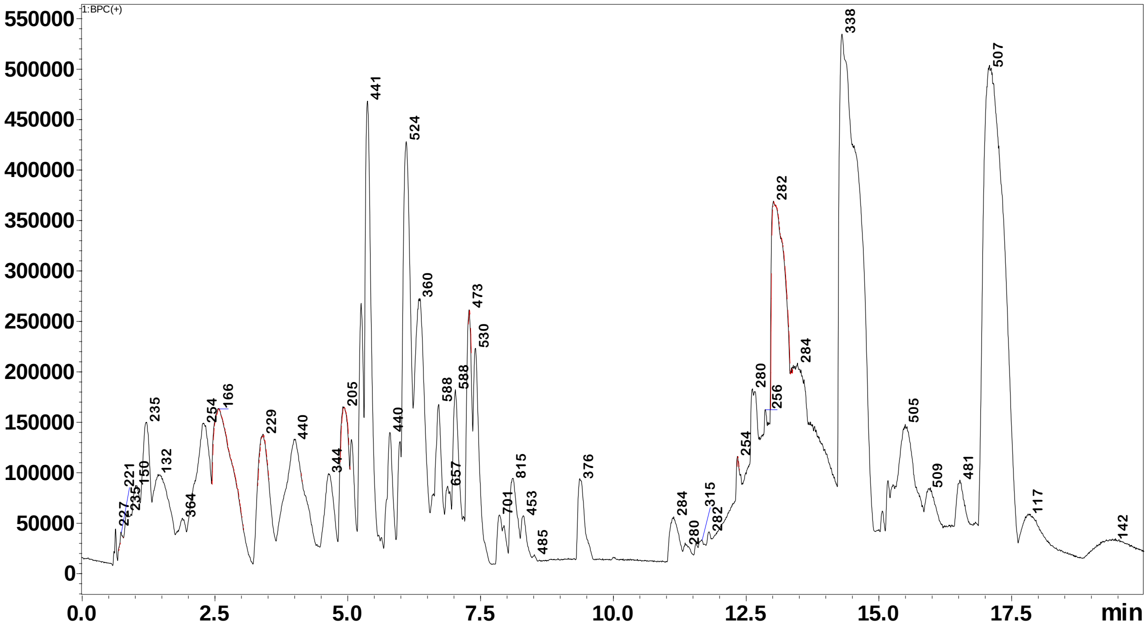

Supplementary Figure 1: The LC-MS chromatograms for crude extracts isolated from BNWU1 (A), BNWU2(B), BNWU4 (C) and BNWU5(D).

(A)

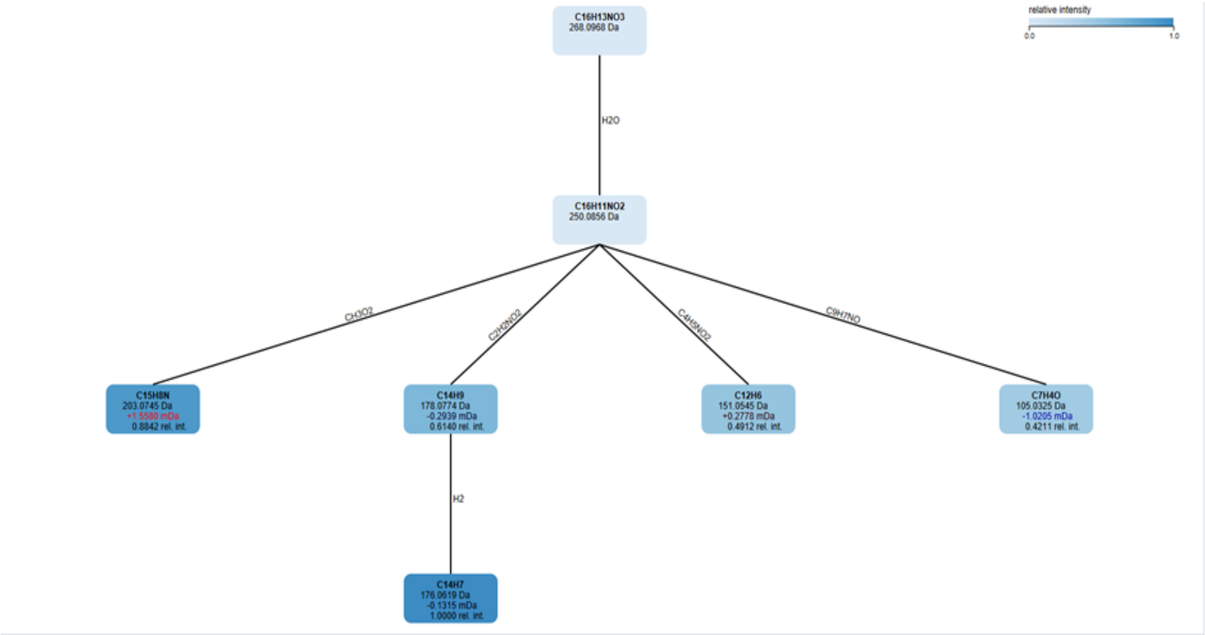

(B)

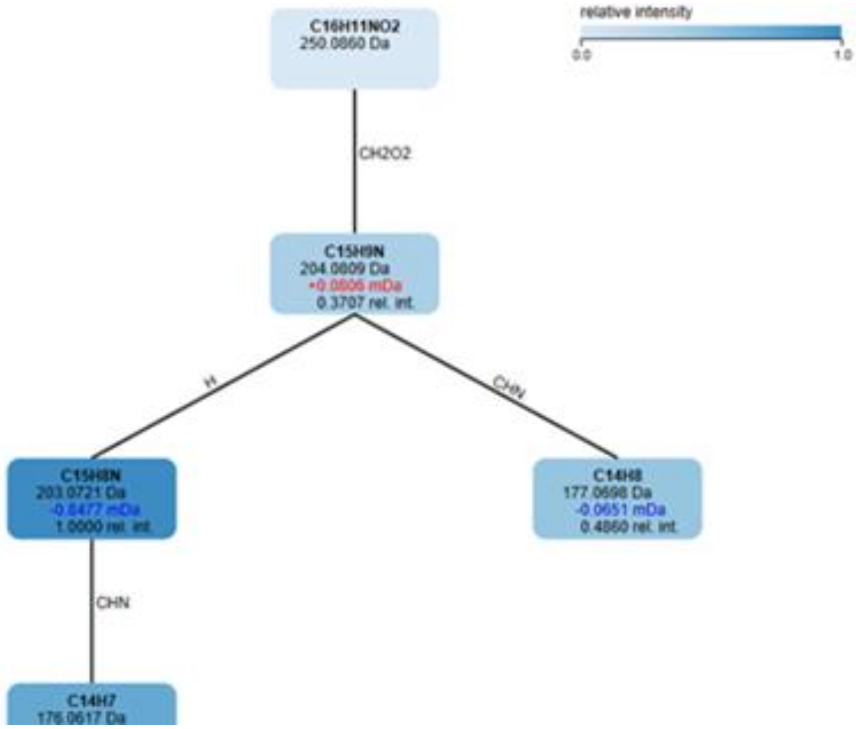

Supplementary Figure 2: Fragmentation pattern for 3-Hydroxy-3-phenacyloxindole (A) and Cincophen (B). Both molecules are listed in Table 3 and share parent mass.
